# Functional diversification of the *GALA* type III effector family contributes to *Ralstonia solanacearum* adaptation on different plant hosts

**DOI:** 10.1111/j.1469-8137.2011.03854.x

**Published:** 2011-12

**Authors:** Philippe Remigi, Maria Anisimova, Alice Guidot, Stéphane Genin, Nemo Peeters

**Affiliations:** 1 INRA, Laboratoire des Interactions Plantes-Microorganismes (LIPM)UMR441, F-31326 Castanet-Tolosan, France; 2 CNRS, Laboratoire des Interactions Plantes-Microorganismes (LIPM)UMR2594, F-31326 Castanet-Tolosan, France; 3 Department of Computer ScienceETH Zurich, Zurich, Switzerland; 4 Swiss Institute of BioinformaticsLausanne, Switzerland

**Keywords:** evolution, functional divergence, host range, *Ralstonia solanacearum*, redundancy, type III effector

## Abstract

- Type III effectors from phytopathogenic bacteria exhibit a high degree of functional redundancy, hampering the evaluation of their precise contribution to pathogenicity. This is illustrated by the GALA type III effectors from *Ralstonia solanacearum*, which have been shown to be collectively, but not individually, required for disease on *Arabidopsis thaliana* and tomato. We investigated evolution, redundancy and diversification of this family in order to understand the individual contribution of the GALA effectors to pathogenicity.
- From sequences available, we reconstructed *GALA* phylogeny and performed selection studies. We then focused on the *GALAs* from the reference strain GMI1000 to examine their ability to suppress plant defense responses and contribution to pathogenicity on three different host plants: *A. thaliana*, tomato (*Lycopersicum esculentum*) and eggplant (*Solanum melongena*).
- The *GALA* family is well conserved within *R. solanacearum* species. Patterns of selection detected on some *GALA* family members, together with experimental results, show that *GALA*s underwent functional diversification.
- We conclude that functional divergence of the *GALA* family likely accounts for its remarkable conservation during *R. solanacearum* evolution and could contribute to *R. solanacearum*’s adaptation on several host plants.

## Introduction

*Ralstonia solanacearum* is a soilborne plant pathogenic bacterium which is able to cause ‘bacterial wilt’ disease on > 200 plant species (Denny, 2006). Soil living bacteria enter the root, colonize xylem vessels and cause wilting of the plant. *R. solanacearum* relies on a type III secretion system (T3SS) and a large repertoire of effectors (T3Es), 74 in the reference strain GMI1000 (Poueymiro & Genin, 2009), to promote pathogenicity.

Although it is frequent among phytopathogenic bacteria that single T3Es are individually dispensable for pathogenicity (Cunnac *et al.*, 2004b; Castaneda *et al.*, 2005; Kvitko *et al.*, 2009), only a few examples of functional redundancy were documented. The best-studied cases include HopM1 and AvrE, located on the CEL locus in *Pseudomonas syringae* pv. *tomato* (*Pst*). Both are able to suppress callose deposition induced after perception of the bacteria by the plant (DebRoy *et al.*, 2004). The double mutant Δ*AvrE*Δ*HopM1* is severely altered in its ability to grow in inoculated plants, while single mutants are not (Badel *et al.*, 2006). Similar functional redundancy was described for AvrPto1 (previously AvrPto) and HopAB2 (previously AvrPtoB) (Lin & Martin, 2005), which target the same receptor-like kinase FLS2 (Gohre *et al.*, 2008; Xiang *et al.*, 2008). Genetic redundancy, through multigene families, can also contribute to functional redundancy. In bacteria, both gene duplication events and lateral gene transfers (LGTs) were shown to drive the appearance of multigene families (Lerat *et al.*, 2005; Treangen & Rocha, 2011). A small number of T3Es are present in a given strain as multimember families. These include *PopP* (three members), *AWR* (five), *SKWP* (six), *HLK* (three) and *GALA* (seven) in *R. solanacearum* and TALs, with up to 28 members present in one *Xanthomonas* sp. strain (Poueymiro & Genin, 2009; Scholze & Boch, 2011). Large effector gene families appear to be more common among the predicted effectors from plant pathogenic fungi and oomycetes (Haas *et al.*, 2009; Stergiopoulos & de Wit, 2009).

As originally stated by Ohno (1970) and later reviewed (Hahn, 2009; Innan & Kondrashov, 2010), when duplicates are retained during evolution, there are three major possible outcomes of gene duplication: neofunctionalization, the evolution of a new function through relaxed selection pressure in one of the two copies; subfunctionalization, the division of ancestral functions among duplicates; and redundancy, the conservation of all functions in both duplicates. The latter is generally explained by the benefit of increased dosage. In functional divergence models (neo- and subfunctionalization), the presence of both copies becomes indispensable because of their new specific functions. To our knowledge, the contribution of these different models to the evolution of effector gene families has never been addressed in plant pathogens.

GALAs are a family of seven effectors in GMI1000 which were initially identified based on their homology with plant F-box proteins (Angot *et al.*, 2006). In eukaryotes, F-box proteins are components of E3-ubiquitin ligases complexes called SCF (SKP1–Cullin1–Fbox) (Hua & Vierstra, 2011). These enzymes target proteins for ubiquitination, leading either to their degradation by the proteasome or to modification of their activity by ubiquitination. The F-box domain mediates the interaction with the SKP1 subunit of the SCF complexes. A protein–protein interaction domain (leucin-rich repeats (LRRs) in the GALAs) enables specific interaction with proteins targeted for ubiquitination. Since bacteria do not possess a proteasome system, it is thought that GALAs could enable *R. solanacearum* to manipulate their host ubiquitin-proteasome system. GALA7 is a host-specificity factor on *Medicago truncatula*, and the F-box domain is required for its function (Angot *et al.*, 2006). On other host plants (*A. thaliana* and tomato), none of the seven single *gala* mutants are affected in their pathogenicity (Cunnac *et al.*, 2004b), whereas the septuple mutant is less pathogenic (Angot *et al.*, 2006). This suggests that, on these plants, at least two different *GALA*s are redundant or have overlapping functions. In this work, we first show that the *GALA* family is well conserved within *R. solanacearum* species, indicating that the maintenance of *GALAs* could be important for bacterial fitness. Then, by comparing synonymous vs nonsynonymous substitution rates in *GALA* coding sequences, we show that *GALAs* are exposed to different patterns of selection. Finally, by combining sequence analysis with experimental data, we provide evidence of functional diversification among *GALAs*. Differential contribution of individual *GALAs* to pathogenicity on several hosts is likely to account for the conservation of this gene family.

## Materials and Methods

### Bacterial strains, growth conditions and plant material

The bacterial strains and plasmids used for this study are described in Supporting Information Table S1. *Escherichia coli* cells were grown in Luria-Bertani (LB) medium at 37°C. *P. syringae* was grown in King's B (KB) medium at 28°C. *R. solanacearum* was grown in complete medium B at 28°C (Plener *et al.*, 2010). Antibiotics were used at the following concentrations (mg l^−1^): rifampicin, 50; spectinomycin, 40; gentamycin, 10; and kanamycin, 50. Plants used in this study were *Arabidopsis thaliana* ecotype Col0, tomato (*Lycopersicum esculentum* cv Marmande VR) and eggplant (*Solanum melongena* cv Zebrina).

### DNA manipulation and genetic constructions

Standard methods were used unless otherwise stated. GMI1000 *GALA* open reading frames (ORFs) were cloned with stop codons using the Gateway system (Invitrogen). *GALA4*, *GALA5*, *GALA6* and *GALA7* were amplified by PCR with Phusion DNA polymerase (Finnzymes, Vantaa, Finland) and BP, gateway-cloned into pDON207 (Invitrogen). The first amplification was performed with specific primers, followed by a second amplification with adaptors oNP291/oNP292 to insert *attB1* and *attB2* sites. Despite several attempts, *GALA2* could not be cloned, probably as a result of intramolecular recombinations. Stop codons were inserted into *GALA1* and *GALA3* pENTRY vectors (Angot *et al.*, 2006) by site-directed mutagenesis, using the QuickChange II XL site-directed mutagenesis kit (Agilent, Santa Clara, CA, USA). GMI1000 *GALA* ORFs were recombined into pEDV6 destination vector (Sohn *et al.*, 2007) using LRII Clonase (Invitrogen). *R. solanacearum gala* mutants were generated by natural transformation using genetic constructions described previously (Cunnac *et al.*, 2004b; Angot *et al.*, 2006). All oligonucleotides used in this study are listed in Table S2. Considering that plasmid-based expression only yields imperfect complementation of mutants in *R. solanacearum* (Angot *et al.*, 2006), we decided not to generate plasmid complementation constructs for the different *gala* mutants.

### Phylogeny, synteny and promoter analysis

The *GALA* sequence dataset was collected from iANT (http://iant.toulouse.inra.fr/bacteria/annotation/cgi/ralso.cgi) and MaGe (http://www.genoscope.cns.fr/agc/microscope/mage/) (Vallenet *et al.*, 2006) web resources. The strains used in this work and their associated phylotype (Fegan & Prior, 2005) are GMI1000 (phylotype I) (Salanoubat *et al.*, 2002), RS1000 (phylotype I) (Mukaihara *et al.*, 2004), IPO1609 (phylotype II) (Guidot *et al.*, 2009), MolK2 (phylotype II) (C. Boucher & S. Genin, unpublished), CFBP2957 (phylotype II), CMR15 (phylotype III) and PSI07 (phylotype IV) (Remenant *et al.*, 2010).

*GALA* sequences were aligned using the PRANK program, which implements the evolution-aware alignment algorithm (Loytynoja & Goldman, 2008), performing well with indel-rich data, as is our case. Phylogenies of all *GALAs* and of individual *GALA* genes were reconstructed using fast maximum likelihood (ML) heuristic search under model LG (Le & Gascuel, 2008) with Γ-rate variation among sites (Yang, 1994), as implemented in PhyMLv3.0 (Guindon *et al.*, 2010). Branch supports were estimated using the new aBayes method, which is fast, accurate and has performance comparable with the Bayesian method (Anisimova *et al.*, 2011).

The synteny analysis was based on data retrieved from MaGe website. The 500 nucleotides upstream of each *GALA* start codon were retrieved and submitted to an automatic search for the presence of *hrp*_*II*_ box (Cunnac *et al.*, 2004a).

### Comparative genomic hybridization

Comparative genomic hybridization of 60 strains spanning the four *R. solanacearum* phylotypes was performed on a microarray under conditions described previously (Guidot *et al.*, 2009). On this microarray, each *GALA* gene was represented by four to five specific 70-nucleotide-long probes (listed in Table S2). A *GALA* gene was considered absent in the tested strain if the base-2 logarithm of the ratio of the normalized hybridization signal with the tested strain over the normalized hybridization signal with the control DNA was lower than the cutoff value of −0.4 for every probe representative of the considered *GALA* gene. This cutoff value was increased compared with previous reports (Guidot *et al.*, 2009) in order to efficiently remove false-positive signals.

### Analyses of selection pressures

Selection pressures on *GALA* were analyzed using Markov models of codon substitution, and likelihood ratio tests (LRTs) were used to detect positive selection (for review see Anisimova & Kosiol, 2009). In these models, selection pressure on the protein is measured by *ω* = *d*_N_/*d*_S_– the ratio of nonsynonymous to synonymous substitution rates (Bielawski & Yang, 2001). Values of *ω* > 1 suggest an effect of positive selection on the protein, whereas values of *ω* < 1 suggest purifying selection of varying degree. For each orthologous *GALA* protein-coding alignment, the likelihood was optimized assuming inferred gene phylogenies. To ensure the robustness of our inferences, we used several models: one-ratio model M0, and site models M3, M1a, M2a, M7, M8 (Yang *et al.*, 2000) and M8a (Swanson *et al.*, 2003). Site models allow selection pressure to vary among sites and are used here to evaluate relative functional importance of different positions in the protein. Models M1a, M7 and M8a may represent the null hypothesis of no positive selection on the dataset, and were compared using LRTs to models M2a, M8 and M8, respectively, to test whether data supported the idea that a significant fraction of sites were under positive selection. The Bayes empirical Bayes (BEB) approach (Yang *et al.*, 2005) was used for classifying sites in each alignment into site categories according to estimated *ω* ratios. The posterior probabilities (PPs) of a site to belong to a particular category were used to evaluate the support of BEB classification. A gene was classified to be under positive selection if at least two LRTs suggested the presence of positive selection.

### Testing for recombination

For each *GALA* alignment package LDhat v2.1 (http://www.stats.ox.ac.uk/~mcvean/LDhat) was used to estimate population recombination rates using the approximate-likelihood coalescent method (Hudson, 2001), and performed recombination tests using the likelihood permutation test (McVean *et al.*, 2002). In addition, Tajima's *D* values (Tajima, 1989) were computed by LDhat. In a classic neutrality test, the null hypothesis of neutral evolution corresponds to *D* = 0. Departures from neutrality may be indicated by significant deviations of *D* from 0. For example, *D* > 0 may be a result of positive (balancing or diversifying) selection or shrinking populations. Values |*D*| > 2 were considered as significant (Tajima, 1989). As standard neutrality tests assume a very simple population model, decoupling the effects of demography and selection is typically nontrivial (Nielsen, 2001).

Finally, for each *GALA* paralog we also estimated selection in the presence of recombination using an approximation to a population genetics coalescent (Wilson & McVean, 2006), as implemented in OmegaMap (http://www.danielwilson.me.uk). In this approach, inference is performed on both parameters simultaneously using the Bayesian method with reversible jump Markov chain Monte Carlo sampling (with 10^6^ generations and discarding 2 × 10^5^ burn-in). Variable models for both selection and recombination parameters were assumed.

### Callose deposition assay

*Pseudomonas syringae* DC3000 overnight cultures were infiltrated in *A. thaliana* leaves at a concentration of 10^8^ cfu ml^−1^. For each strain, three leaves were infiltrated on three plants. Leaves were harvested after 24 h and callose was stained with aniline blue as described (Adam & Somerville, 1996). Leaf samples were mounted in 50% glycerol and observed with a Zeiss Axiophot II epifluorescence microscope (365 nm BP excitation filter, 395 nm chromatic beam splitter, 397 nm LP emission filter). Between five and 15 independent images (*c.* 1 mm^2^) were taken for each strain and subsequently analyzed with ImageJ (Rasband, 1997). A two-tailed Mann–Whitney statistical test was performed with Prism, version 5.00 (GraphPad Software, La Jolla, CA, USA).

### *Ralstonia solanacearum* pathogenicity assays, statistical analysis

Tomato and eggplant pathogenicity assays were performed by watering *c.* 5- to 6-wk-old plants with 50 ml of a bacterial suspension containing 10^7^ cells ml^–1^. *A. thaliana* plants were inoculated with a bacterial suspension at the same concentration, as described by Deslandes *et al.* (1998). The plants were incubated in a growth chamber at 28°C for tomato and eggplant (14 h light : 10 h dark) or 16 h at 27°C (light) : 8 h at 26°C (dark) for *A. thaliana*. Disease development was scored daily, using a macroscopic scale describing the observed wilting: 1 for 25% of the leaves wilted; 2 for 50%; 3 for 75% and 4 for complete wilting. For subsequent analysis the data was transformed into a binary index: 0 for < 50% of wilted leaves and 1 for more or equal to 50% wilted leaves. To compare the disease development of two given strains, we used the Kaplan–Meier survival analysis (Bland & Altman, 1998) with the Gehan–Breslow–Wilcoxon method to compute the *P*-value to test the null hypothesis of identical survival experience of the two tested strains. A *P*-value smaller than 0.05 was considered significant. Statistical analyses were done with Prism version 5.00 (GraphPad Software).

## Results

### The *GALA* family is conserved in the *R. solanacearum* species complex

**Fig. 1:**
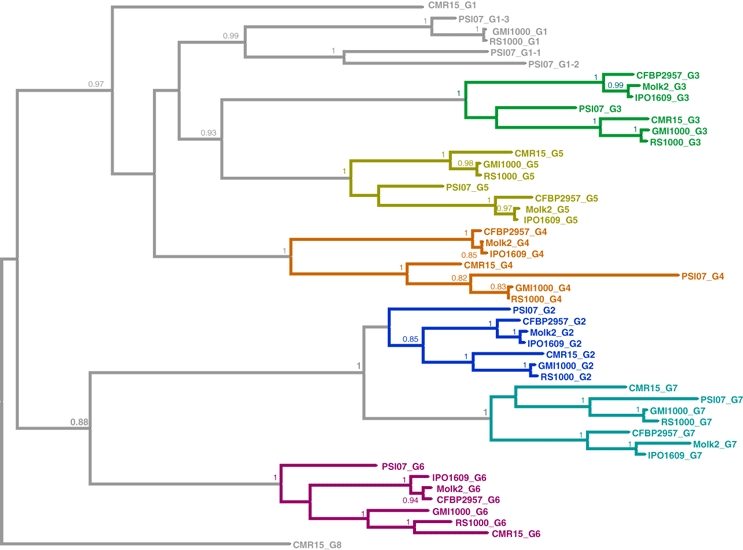
*GALA* phylogeny. Unrooted maximum-likelihood phylogenetic reconstruction of *GALAs* from strains GMI1000, RS1000, CMR15, IPO1609, Molk2, CFBP2957 and PSI07. Branch support values (aBayes) are shown only for clades with strong support (> 0.9).

The *R. solanacearum* species complex exhibits a high degree of genetic diversity and is structured in four monophyletic groups, termed phylotypes. These groups correspond to specific geographic distributions (Fegan & Prior, 2005). Full genome sequences from seven strains, representative of the whole species diversity, are now available. These include the reference strain GMI1000 (Salanoubat *et al.*, 2002) from phylotype I, draft sequences of phylotype II strains IPO1609 (Guidot *et al.*, 2009) and MolK2 (C. Boucher & S. Genin, unpublished) and the newly sequenced strains CFBP2957, CMR15 and PSI07 from phylotypes II, III and IV, respectively (Remenant *et al.*, 2010). *GALA* sequences were retrieved from these complete genomes (see Table S3 for accession numbers). A genome sequence from strain UW551 is also available (Gabriel *et al.*, 2006) but we did not use it since its *GALA* sequences are exactly identical to IPO1609. Moreover, seven *GALAs* from RS1000, a phylotype I strain (Mukaihara *et al.*, 2004), were included in the analysis. Between six (in MolK2, IPO1609) and nine (in PSI07) *GALA*s were detected in these strains. Using this dataset, we reconstructed the *GALA* phylogeny in the *R. solanacearum* species complex (Fig. 1). *GALAs* cluster by orthologous groups, indicating that the ancestral strain most likely had at least seven *GALAs* (namely *GALA1*, *2*, *3*, *4*, *5*, *6*, *7*), which subsequently evolved independently within each lineage. Two subclades of paralogs could be defined with high support: *GALA2*, *6*, *7* and *GALA1*, *3*, *4*, *5*. Using a large (60) and diverse set of *R. solanacearum* strains, we performed comparative genomic hybridization (CGH) on a dedicated microarray harboring T3Es probes designed on the sequenced GMI1000, IPO1609 and Molk2 strains (Guidot *et al.*, 2009). We were able to detect the presence of *GALA4* in all strains tested, the others, *GALA2*, *3*, *5*, *6* and *7*, being present in most strains with some exceptions (Table S4). The four *GALA1* probes do not hybridize with DNA extracted from any of the 35 phylotype II strains or any of the 13 phylotype III strains, indicating that *GALA1* is either absent or highly divergent in these phylotypes. Based on the fact that *GALA1* is present in CMR15, the sequenced phylotype III strain, and absent from the sequenced phylotype II strains IPO1609, Molk2 and CFBP2957, we hypothesize that *GALA1* is indeed absent in phylotype II and present but divergent in the other phylotype III strains.

Since *GALAs* are present in all sequenced strains, we scrutinized the loci of these different *GALAs* in the sequenced strains. *GALA4* and *GALA5* are in operon, while *GALA6* and *GALA7* are in direct tandem repeat. Together with *GALA2*, these three loci are well conserved in all sequenced strains (Fig. S1), strengthening the hypothesis that those genes were already present in the common ancestor of these strains. The *GALA3* loci are more variable, with many ‘inserted genes’ in the phylotype II and IV strains compared with GMI1000. The strain PSI07 is somewhat peculiar since it possesses three copies of *GALA1* (*GALA1-1*, *GALA1-2* and *GALA1-3*) and *GALA2* is found in the close vicinity of *GALA1-3*. Furthermore, only the 100 last amino acids of *GALA4* from this strain are conserved and *GALA1-3* is truncated after the first 200 amino acids.

In GMI1000, all *GALAs* but *GALA1* are regulated by HrpB, the general regulator of T3SS and T3Es (Cunnac *et al.*, 2004a). This regulation is associated with the presence of the *hrp*_*II*_ box domain in the promoters of *GALA2*, *GALA3*, *GALA4*, *GALA6* and *GALA7* (Cunnac *et al.*, 2004a). Promoter analysis of *GALA* genes from all sequenced strains indicates that *GALAs* (except for *GALA1* and *GALA5*, which is in operon with *GALA4*) have a conserved *hrp*_*II*_ box domain (Table S3). Unexpectedly, *GALA1-1*_PSI07_ also has a *hrp*_*II*_ box sequence in its promoter. The distance between the position of the *hrp*_*II*_ box and the start codon is well conserved in GMI1000 *GALA* promoters (*c.* 100 bp) but is greater, *c.* 400 bp, in *GALA4*_*IPO1609*_, *GALA4*_*CMR15*_, *GALA6*_*CFBP2957*_ and *GALA3*_*PSI07*_. This finding suggests that HrpB-regulation of most *GALAs* may be conserved in all *R. solanacearum* strains.

### Differential selection pressures in *GALA* paralogs

We then investigated the patterns of selection acting on the *GALAs.* Positive selection was detected in *GALA3*, *4*, *6* and *7* (Table 1) using at least two likelihood ratio tests (LRTs) for positive selection (see the Materials and Methods section). By contrast, no evidence of positive selection was found on *GALA1*, *2* and *5* (Table 1). The largest fraction of strictly conserved sites (57%) was found in *GALA5*, while the largest fraction of neutrally evolving sites (63%) was found in *GALA2* (Table 1). *GALA7* experienced most sites under positive selection (8%).

**Table 1:** Selection pressures in *GALA* paralogs

					*P*-values for LRTs comparing codon models^a^				Proportions of sites in different selection regimes^b^			
						
*GALA* copy	Number of strains	Alignment length (nt)	Population recombination rate, *N*_*e*_*r* (*P*_LPT_)^a^	Tajima's *D*^a^	M0 vs M3	M1a vs M2a	M7 vs M8	M8a vs M8	Strict negative (%) (*ω* < 0.15)	Relaxed negative (%) (0.15 < *ω* < 0.9)	Neutral (%) (0.9 < *ω* < 1)	Positive (%) (*ω* = 1)
*GALA1*	6	2046	0.00 (1.00)	**2.1**	**0.000**	1.000	**0.045**	0.256	42 (*ω* = 0.08)	25 (*ω* = 0.31)	33	0
*GALA2*	7	3237	0.00 (1.00)	1.7	**0.000**	1.000	**0.022**	0.187	32 (*ω* = 0.04)	4.5 (*ω* = 0.25)	63	0
*GALA3*^c^	9	1971	**12.25** (**0.003**)	**4.2**	**0.000**	**0.023**	**0.001**	**0.000**	28 (*ω* = 0.05)	57 (*ω* = 0.53)	10	5 (*ω* = 3.28)
*GALA4*^c^	8	1677	**14.29** (**0.001**)	**3.5**	**0.000**	**0.040**	**0.002**	**0.002**	39 (*ω* = 0.06)	58 (*ω* = 0.49)	0	3 (*ω* = 5.90)
*GALA5*	8	1683	0.00 (0.000)	**2.1**	**0.000**	1.000	0.170	0.457	57 (*ω* = 0.06)	19 (*ω* = 0.24)	24	0
*GALA6*^c^	9	1866	**6.12** (**0.000**)	**4.2**	**0.000**	0.653	**0.035**	**0.048**	48 (*ω* = 0.06)	48 (*ω* = 0.54)	0	4 (*ω* = 2.58)
*GALA7*^c^	9	2052	**16.33** (**0.000**)	**4.3**	**0.000**	**0.000**	**0.000**	**0.000**	46 (*ω* = 0.05)	28 (*ω* = 0.51)	18	8 (*ω* = 3.7)

a Significant values for selection, recombination and neutrality tests are shown in bold.

b Estimates of selection regimes are reported according to the model M8 if the likelihood ratio test (LRT) comparing M8a and M8 was significant. Otherwise, selection regimes are reported according to model M8a. For strict and relaxed negative selection, the average *ω* -value over respective selection classes (with either *ω* < 0.15 or 0.15 < *ω* < 0.9) is shown. In each of four selection categories shown, the highest frequency value of sites is underlined.

c *GALA*s where positive selection was detected using at least two LRTs for positive selection. Note that the LRT comparing M0 and M3 is not a test for positive selection but for variability of selection pressure among sites.

Given the inferred phylogeny of the *GALAs*, it is interesting to note that both *GALA* subclades (*GALA2*, *6*, *7* and *GALA1*, *3*, *4*, *5*) evolve in a similar fashion. Namely, two *GALAs* in each subclade (*GALA6*, *7* and *GALA3*, *4*) are affected by positive selection, whereas at least one *GALA* in each clade (*GALA2* and *GALA1*, *5*) is mostly conserved. Of particular interest are the closely related *GALA3* and *GALA5*, which originated from a recent duplication event, as shown in Fig. 1. *GALA3* contains sites under positive selection and is generally evolving under relaxed purifying selection, but in *GALA5* the majority of sites are very conserved and there are no sites under positive selection (Table 1). Similarly, the closely related *GALA2* and *GALA7* have very different patterns of selection: while *GALA7* has a relatively large fraction of sites (8%) affected by strong positive selection, *GALA2* mostly evolves neutrally (63%) and has no site under positive selection. In addition, compared with other *GALA* genes, *GALA2* and *GALA5* have a much larger fraction of sites (22 and 23%, respectively) that are very strictly conserved, with the ML estimates of *ω* < 0.1 and the posterior estimate of *ω* < 0.1. By contrast, *GALA3*, *GALA4*, *GALA6* have none and *GALA7* has only very few such sites (< 1%). Overall, such striking differences in patterns of selection between *GALAs* are indicative of functional divergence.

### Interplay between selection and recombination

The presence of a high degree of recombination can hamper LRTs for positive diversifying selection, leading to elevated rates of false positives (Anisimova *et al.*, 2003). However, inference of recombination may also be affected by selection forces (Reed & Tishkoff, 2006; O'Reilly *et al.*, 2008).

In an attempt to disentangle the signatures of selection and recombination in our data, we performed tests for recombination and simultaneous inference of selection and recombination parameters. The population recombination rate *N*_*e*_*r* was estimated to be > 0 for *GALA3*, *4*, *6*, and *7*, with significant likelihood permutation tests (*P*_LPT_ < 0.05; Table 1). It is exactly for these *GALA* paralogs that strong positive selection was detected. However, our estimates of population recombination rates appear to be very low, and would be unlikely to affect our inferences of selection (Anisimova *et al.*, 2003). Yet, judging by estimates from Table 1 and the occasional deviations from *R. solanacearum* phylogeny within each *GALA* clade (Fig. 1), it is more likely that a combination of both recombination and different types of selection (diversifying and directional) may be shaping GALA proteins. Indeed, inference of selection in the presence of recombination using OmegaMap confirmed that both positive selection and a low degree of recombination operate on GALA proteins. The amount of selection inferred by our codon models was consistent with estimates from OmegaMap, and with Tajima's *D* (Table 1).

### GALA4, but not GALA5, interferes with callose deposition

**Fig. 2:**
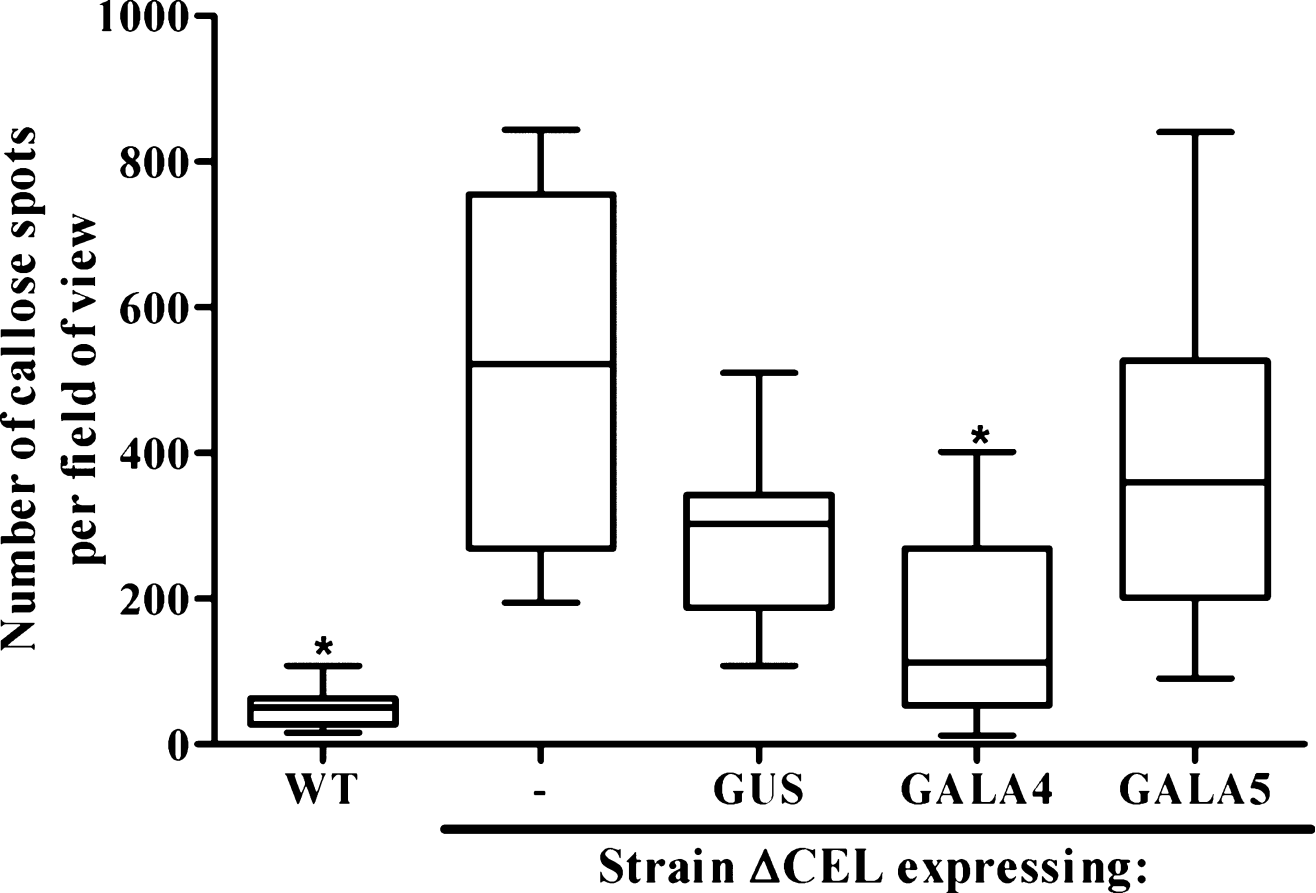
GALA4 but not GALA5 can interfere with callose deposition. *Arabidopsis thaliana* leaves were inoculated with *Pseudomonas syringae* strains and stained for callose 24 h after infection. A box plot diagram of papillae number from at least five independent fields of view is shown for each strain. The horizontal black bar is the median and the boxes indicate lower and upper quartiles. This experiment was repeated four times with similar results. *, median number of callose spots per field of view in the tested strain is statistically different from the strain *Pst* DC3000 ΔCEL (Mann–Whitney test, *P* < 0.05). WT, wild-type.

An increasing number of studies show that many, if not most, T3Es are able to suppress PAMP (Pathogen-associated molecular pattern)- or Effector-Trigerred Immunity (PTI and ETI, respectively) (Hann *et al.*, 2010; Block & Alfano, 2011). We could not detect any effect of *GALA* expression on AvrRpm1-triggered immunity (result not shown). In order to test if GALAs are able to interfere with PTI, we took advantage of the pEDV6 vector (Sohn *et al.*, 2007) to express *GALA*s from *P. syringae* DC3000 strain ΔCEL (Alfano *et al.*, 2000). This strain, with deleted HopM1 and AvrE T3Es, is no longer able to suppress callose papillae at the cell wall, a basic defence mechanism elicited during PTI. The pEDV6 vector enables expression of GALAs fused to the N-terminus of the *P. syringae* effector AvrRPS4 (Sohn *et al.*, 2007). This N-terminal extension allows effective translocation of the chimeric T3E into plant cells and later cleavage to release the effective full-length GALA effectors in the plant cell. We monitored callose deposition in *A. thaliana* leaves after infiltration with *P. syringae* strain ΔCEL expressing each GALA. *P. syringae*-dependent delivery of GALA1, GALA3, GALA6 and GALA7 leads to a weak decrease in callose deposition but was not statistically significant (data not shown). *P. syringae*-dependent delivery of GALA4 reproducibly decreased the median number of callose spots per field of view, whereas this effect was not observed with GALA5 (Fig. 2). This latter observation is not because of the absence of GALA5 translocation since we could detect the cleaved full-length GALA5 by western blot (Sohn *et al.*, 2007) after infiltration into *A. thaliana* leaves (data not shown). Surprisingly, the expression of GALA4 in *P. syringae* strain ΔCEL did not increase bacterial multiplication in *A. thaliana* leaves (data not shown), as is usually the case with PTI-suppressing effectors (Nomura *et al.*, 2006; Sohn *et al.*, 2007). The fact that callose suppression mediated by GALA4 is weaker than observed in the aforementioned studies may explain this result.

### Differential *GALA* requirement for pathogenicity on *A. thaliana,* tomato and eggplant

**Fig. 3:**
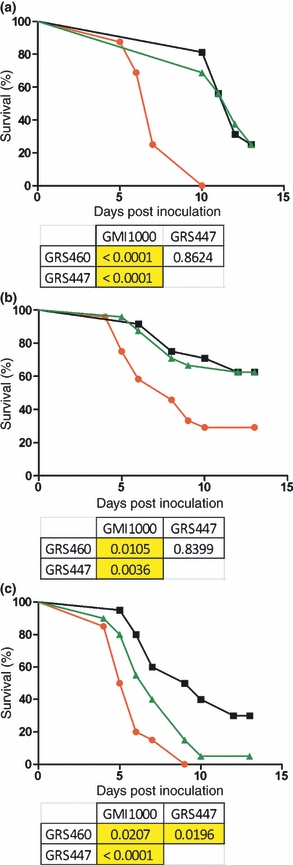
*gala2*, *3*, *6*, *7* mutant (strain GRS460, triangles) phenocopies the septuple *gala* mutant (strain GRS447, squares) on *Arabidopsis thaliana* and *Lycopersicon esculentum* (tomato) but not on *Solanum melongena* (eggplant). Kaplan–Meier survival analysis of *A. thaliana* (a), tomato (b) and eggplant (c) plants inoculated with *Ralstonia solanacearum*. GMI1000, wild-type, circles. Each strain was inoculated on 16 *A. thaliana* plants (a), 24 tomato plants (b) and 20 eggplant plants (c). Correspondence between strains and color code is conserved in the three graphs. *P*-values from Gehan–Breslow–Wilcoxon tests are associated with each graph. Yellow boxes indicate a *P*-value of <0.05. These experiments were performed at three times with similar results.

**Fig. 4:**
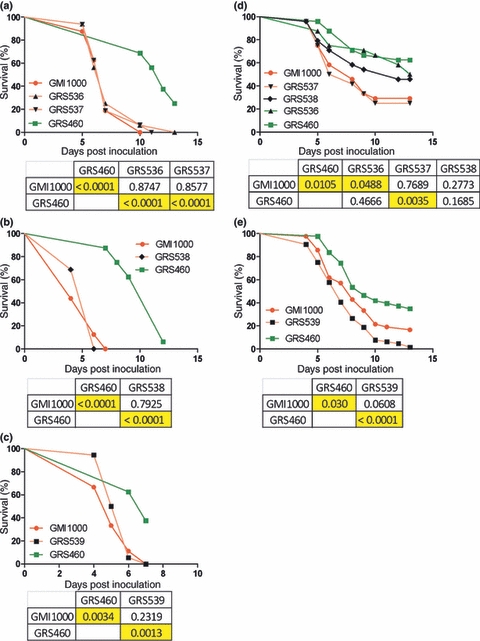
Pathogenicity assays of *Ralstonia solanacearum* triple *gala* mutants on *Arabidopsis thaliana* and *Lycopersicon esculentum* (tomato). Kaplan–Meier survival analysis of *A. thaliana* (a, b, c) and tomato (d, e) plants inoculated with *R. solanacearum* mutants. Genotypes of the tested strains are the following: GMI1000, wild-type; GRS536, *gala2*, *3*, *7*; GRS537, *gala2*, *3*, *6*; GRS538, *gala3*, *6*, *7*; GRS539, *gala2*, *6*, *7*; GRS460, *gala2*, *3*, *6*, 7. Each strain was inoculated on 16 *A. thaliana* plants (a, b, c) and 24 tomato plants (d, e). *P*-values from Gehan–Breslow–Wilcoxon tests are associated with each graph. Yellow boxes indicate a *P*-value of <0.05. These experiments were performed three times with similar results.

We previously showed that *GALAs* are collectively required for pathogenicity on tomato and *A. thaliana*, and that *GALA7* is a host-specificity factor on *M. truncatula* (Angot *et al.*, 2006). In order to identify the contribution of each *GALA* to the pathogenicity of *R. solanacearum* GMI1000, we generated strains carrying various combinations of *GALA* gene disruptions and assayed them for their pathogenicity on *A. thaliana*, tomato and eggplant. We identified strain GRS460 (*gala2*, *3*, *6*, *7*) as phenocopying the septuple mutant GRS447 on *A. thaliana* and tomato (Fig. 3a,b). This strain has an intermediate phenotype on eggplant, significantly different from both GMI1000 and GRS447 (Fig. 3c). To identify which of *GALA2*, *3*, *6* or *7* were required for pathogenicity on *A. thaliana* and tomato, we generated the four triple mutants, GRS536 (*gala 2*, *3*, 7), GRS537 (*gala 2*, *3*, 6), GRS538 (*gala 3*, *6*, *7*) and GRS539 (*gala 2*, *6*, *7*). On *A. thaliana*, these four triple mutants have a wild-type phenotype, indicating that *GALA2*, *3*, *6* and *7* are all functional and redundant on *A. thaliana* (Fig. 4a,b,c). Indeed, the presence of any of them, in conjunction with the still present *GALA1*, *4*, and *5*, is sufficient to promote pathogenicity at a wild-type level. On tomato, GRS536 (*gala2*, *3*, *7*) phenocopies GRS460, indicating that *GALA6* has no significant virulence effect in a *GALA1*, *4*, *5* background (Fig. 4d). Conversely, GRS537 (*gala2*, *3*, *6*) and GRS539 (*gala2*, *6*, *7*) have a wild-type phenotype, indicating that *GALA7* and *GALA3* are functional on tomato (Fig. 4d,e). The phenotype of strain GRS538 (*gala3*, *6*, *7*) is intermediate and disease kinetics induced by this strain is not statistically different from either GRS460 or GMI1000 (Fig. 4d). *GALA*2 may therefore have a weak virulence effect on tomato, not sufficient to restore full pathogenicity in a *GALA1*, *4*, *5* background.

Altogether, our data show that different subsets of *GALA*s promote disease on different hosts, providing additional evidence of functional diversification of the *GALA* family.

## Discussion

Evolution of type III effectors is shaped by strong selection pressures, either to escape recognition by the plant immune system or to gain efficiency to promote bacterial multiplication within one or several host plants. T3Es are exchanged between strains by LGT at relatively high frequency or can adapt to optimize their function via pathoadaptation (Ma & Guttman, 2008; Arnold & Jackson, 2011). Those two mechanisms are thought to be responsible for much of the variation observed in T3Es complements from phytopathogenic bacteria, in terms of both presence/absence and allelic variations. Here we describe an unusual fate for T3Es genes, that is, creation of a multigene family via several rounds of duplications and subsequent retention of six to nine family members across the *R. solanacearum* species complex.

Since very few bacterial species have F-box proteins (Angot *et al.*, 2007) and considering that *GALA2*, *3*, *4*, *5*, *6* and *7* have a common ancestor, it is very likely that those *GALAs* are of monophyletic origin, as suggested previously (Kajava *et al.*, 2008). The more variable presence of *GALA1* could be explained by additional LGT events or by multiple independent losses. This could also apply to *GALA8*, a newly identified *GALA* in strain CMR15 (Remenant *et al.*, 2010).

The inferred *R. solanacearum* phylogeny and good synteny conservation support vertical inheritance among *GALA* paralogs. From our phylogeny, synteny and CGH analyses, we conclude that at least seven *GALA* genes were already present in the ancestral strain and have subsequently evolved within *R. solanacearum* genome for a long time. The *GALA* T3E family, as a whole, can therefore be considered as a ‘core’ pathogenicity determinant in the *R. solanacearum* species complex. Conversely, the family of TAL T3Es expanded greatly in *Xanthomonas* sp. (up to 28 members per strain), but only in some lineages (Scholze & Boch, 2011). This suggests a more plant-specific requirement of these T3Es as opposed to the seemingly ubiquitous use of the GALAs.

In order to understand which selection forces were responsible for *GALA* conservation, we conducted statistical sequence analyses followed by functional description of these type III effectors. Our bioinformatics analyses suggest that both recombination and selection operate on GALA proteins. A low degree of recombination appears to be coupled with strong positive selection on *GALA* 3, 4, 6, and 7. Neither selection nor recombination can be detected on *GALA* 1, 2, and 5.

Two patterns of selection can be described. *GALA3*, *GALA4*, *GALA6* and *GALA7* show signs of diversifying selection while *GALA1*, *GALA2* and *GALA5* do not. The differences of selection pressures between *GALA*s are strongly indicative of a functional divergence evolutionary scenario. We therefore performed additional experiments to test this hypothesis. GALA4 significantly decreases callose deposition elicited by *P. syringae* DC3000 strain ΔCEL, while the closely related GALA5 did not. Putative E3-ubiquitine ligase function of GALA proteins is in part dependent on their ability to interact with SKP1-like proteins, which has previously been characterized (Angot *et al.*, 2006). GALA4 is unable to interact, in yeast-two-hybrid, with any member of the *Arabidopsis* SKP1-like (ASK) family, probably because of a variation in a conserved residue in the F-box domain (Angot *et al.*, 2006). GMI1000 GALA4’s function is therefore probably independent of any E3-ubiquitine ligase activity, as has already been reported for other F-box proteins (Galan *et al.*, 2001). Other GALAs exhibited different interaction specificities for ASK and MSK (*Medicago* SKP1-like) proteins, in a way reminiscent of plant F-box proteins (Risseeuw *et al.*, 2003; Angot *et al.*, 2006). Although the significance of these interaction differences is not known, this suggests variations within F-box domains of ASK-binding GALAs. However, these variations are not thought to affect the general ability of the GALAs to be part of SCF complexes, because GALA1, 3, 5, 6 and 7 are all able to interact with some ASKs/MSKs. Finally, pathogenicity assays with *gala* polymutants enabled us to uncover plant-specific functions for some *GALAs*. Contrary to the situation on *A. thaliana*, *GALA3* and *GALA7* are not redundant with *GALA6* on tomato. The presence of *GALA1*, *4* and *5* is sufficient to increase pathogenicity on eggplant, but not on tomato or *A. thaliana*. Differential *GALA* requirement on tested host plants indicates that *GALAs* contribute to pathogenesis on these hosts. This is further confirmed by *GALA7* which is required to extend *R. solanacearum*’s host range to *M. truncatula* (Angot *et al.*, 2006). Interestingly, among the positively selected sites of *GALA7*, 66% are in the LRR region and 92% of the sites present in an LRR are exposed to the solvent, according to the previously predicted structure (Kajava *et al.*, 2008). This could suggest an adaptation of *GALA7* to interact with specific *M. truncatula* target proteins. Divergence in substrate recognition during evolution of F-box proteins has been documented in the case of the TIR1/AFB family of auxin receptors (Parry *et al.*, 2009). Hence, even if some *GALAs* perform partially redundant functions during infection, several lines of evidence clearly show that the *GALA* gene family underwent diversification at the protein-coding level.

Functional diversification occurs either by subfunctionalization or by neofunctionalization. During subfunctionalization, the rate of evolution of the two copies is expected to be symmetrical (Innan & Kondrashov, 2010). Inversely, during neofunctionalization, one copy evolves faster and acquires a new function while the other maintains its original function. We have shown that *GALAs* are organized into two subclades: *GALA1*, *3*, *4*, *5* and *GALA2*, *6*, *7*. In each of these subclades, at least one *GALA* experienced purifying and neutral selection (*GALA2* and *GALA1*, *GALA5*) while the two others in each subclade underwent diversifying positive selection. This is consistent with a neofunctionalization model in which *GALA2* and *GALA5* (and/or *GALA1*) would have kept the ‘ancestral’ function, while *GALA6* and *GALA7*, on one hand, and *GALA*3 and *GALA*4 on the other hand, could have acquired new functions through diversifying selection. The functional analysis provides data to support this model. Indeed, we show that GALA5 does not affect callose deposition. By parsimony principle, this may indicate that GALA4 acquired this ability in the course of divergence from the ancestral allele. Pathogenicity assays on tomato show that *GALA7* is different from *GALA6* by its requirement for full pathogenicity when associated with *GALA1*, *4* and *5*. Furthermore, *GALA7* evolved essential pathogenicity function on *M. truncatula*, not shared by any other *GALA*s (Angot *et al.*, 2006).

Overall, our work shows that the *GALA* gene family underwent functional diversification, possibly via neofunctionalization, which resulted in specific adaptation of some *GALA*s on different host plants. Given *R. solanacearum*’s wide host range, these specificities likely provided the selective advantage responsible for the remarkable conservation of the *GALAs* in the entire species. A similar mode of evolution was recently described for the ROP5 family of injected pseudokinases from *Toxoplasma gondii* and was proposed to contribute to the ability of this parasite to infect a wide host range (Reese *et al.*, 2011). Hence, duplications of important pathogenicity genes may represent a general mechanism providing pathogens with the potential to adapt to a large host spectrum by evolving new functions.

## Supporting Information

**Fig. S1** Synteny in *GALAs*' loci.

**Table S1** List of strains and plasmids used in this study

**Table S2** List of oligonucleotides used in this study

**Table S3** *GALA* accession numbers andposition of *hrp_II_* box

**Table S4** Comparative genomic hybridization analysis
