# Supplementary material for: Functional diversification of the GALA type III effector family contributes to Ralstonia solanacearum adaptation on different plant hosts
Source: New Phytol. 2011 Dec;192(4):976–87. doi: 10.1111/j.1469-8137.2011.03854.x (PMC3263339; doi:10.1111/j.1469-8137.2011.03854.x)
Supplement: Supplementary file 2 [file nph0192-0976-SD2.pdf]

Table S1: Bacterial strains and plasmids used in this study

**Strains**		
Escherechi coli DH5α	F^- *recA lacZ* DM15	Bethesda Research Laboratory
Ralstonia solanacearum		
GMI1000	Wild-type strain	(Boucher *et al.* , 1987)
GRS447	Δ*gala2,4,5,6,7* , *gala1*:: Ω, *gala3*:: Ω, Gm^r Spc^r Kan^r	(Angot *et al.* , 2006)
GRS460	Δ*gala2,6,7* , *gala3*:: Ω,Spc^r	(Angot *et al.* , 2006)
GRS536	Δ*gala2* , *gala3*:: Ω, *gala7* ::pCZ367,Spc^r Gm^r	This work
GRS537	Δ*gala2* , *gala3*:: Ω, *gala6* ::pCZ367,Spc^r Gm^r	This work
GRS538	Δ*gala6,7* , *gala3*:: Ω, Spc^r	This work
GRS539	Δ*gala2,6,7* , Kan^r	This work
Pseudomonas syringae pv. *tomato*		
DC3000	Wild-type strain, Rif^r	
DC3000 ΔCEL	Δ*avrE-shcN* , Rif^r Spc^r	(Alfano *et al.* , 2000)
**Plasmids**		
pCZ367	pUC18-derived vector used for insertional mutagenesis of *gala6* and *gala7*	(Cunnac *et al.* , 2004b)
pEDV6	pAvrRPS4::AvrRPS4$_{1\text{-}136}$-HA-GW	(Sohn et al., 2007; Fabro *et al.* , Submitted)
pPR181	pENTRY-GALA1*	This work
pPR156	pENTRY-GALA3*	This work
pPR126	pDON207-GALA4*	This work
pPR134	pDON207-GALA5*	This work
pPR122	pDON207-GALA6*	This work
pNP200	pDON207-GALA7*	This work
pPR179	pEDV6-GALA1*	This work
pPR180	pEDV6-GALA3*	This work
pPR136	pEDV6-GALA4*	This work
pPR137	pEDV6-GALA5*	This work
pPR124	pEDV6-GALA6*	This work
pPR103	pEDV6-GALA7*	This work

[a]Spc^r, Gm^r, Kan^r, Rif^r = resistant to spectinomycin, gentamycin, kanamycin and rifampicin

Reference

Boucher CA, Barberis PA, Trigalet AP, Demery DA. 1985. Transposon mutagenesis of Pseudomonas solanacearum: isolation of Tn5-induced avirulent mutants.Journal of General Microbiology 131: 2449-2457

Table S2: Oligonucleotides used in this study

**GALA cloning**		
oNP572	GGAGATAGAACCATGGGATACCACTTCTGGGCCG	5'GALA4
oNP573	CAAGAAAGCTGGGTCTCMCGTCTTGAGCACGG	3'GALA4
oNP574	GGAGATAGAACCATGAAACCTGTCGCTCCGGTTGG	5'GALA5
oNP575	CAAGAAAGCTGGGTCTCMGTGCCGCATCGTCA	3'GALA5
oNP576	GGAGATAGAACCATGGTGTCGCGCATTCCCACC	5'GALA6
oNP577	CAAGAAAGCTGGGTCTCMGTCCCTCCACCGCA	3'GALA6
oNP526	GGAGATAGAACCATGATGTTCAAGCGCATCGAC	5'GALA7
oNP527	CAAGAAAGCTGGGTCTCMACCCCGGTACGAG	3'GALA7
oNP291	GGGGACAAGTTTGTACAAAAAAGCAGGCTTCGAAGGAGATAGAACCATG	5' adaptor with attB1
oNP292	GGGGACCACTTTGTACAAGAAAGCTGGGTC	3' adaptor with attB2
oPR101	ACGTTCCATTCCCTGTAGGGTGGGCGCG	5'GALA1 STOP site-directed mutagenesis
oPR102	CGCGCCCACCCTACAGGGAATGGAACGT	3'GALA1 STOP site-directed mutagenesis
oPR103	ACGCTGCGGATTTAGGGTGGGCGCGCCG	5'GALA3 STOP site-directed mutagenesis
oPR104	CGGCGCGCCCACCCTAAATCCGCAGCGT	3'GALA3 STOP site-directed mutagenesis
**CGH microarray probes**		
RSp0914	CGGCGAACAGATCGGAGACCTGGCATGGCAAGAGTTGATGGCATCGGTTGACCGGAGCGGCATGACGTTC	probe GALA1
RSp0914A	TACCAAGACCTTGCACAGGATGCGCGCCGTCAGCAAGACCCTGAAGGCGGCGGCAGACACCGAGATCCG	probe GALA1
RSp0914B	TCGATCTCGGCTTCAACGCCATCGGCGATGAGGGGCTGCAAGCACTGGCCACCAACACCTCGCTCACCTC	probe GALA1
RSp0914C	AAGCACTGGCTGCCAATCGCAGCCTGACCAAGCTCAATATCAGCCAGAATGACATCCGGTCCGCGGGCGC	probe GALA1
RSp0672	CGGGAAACCAGGTGGAGCAAGACGGTTACGACAAGACCGCCAACAACATCACCGCGCAAGGGGCCTGGGC	probe GALA2
RSp0672A	GCGCCAAATCGTTCCGGGCGATCGCGTATCTCGCCGGCTTGCCGCTGGAGTCGCTCAATGTGGCGGG	probe GALA2
RSp0672B	GCCGTCGGTGCGGCACCTGGACCTGAGCGGATGCACCGGCTCCGCGGTCTCGGAGGCGGGCCTGGCT	probe GALA2
RSp0672C	CGGAGGCTGGCGGCAAACCGCACGCTCGTCTCGCTGGACCTGCGCGGCAACACCATCGACGTCGCCG	probe GALA2
RSp0028	CGCCTCGTTGGACGTGGGTGCCAACAAGCTGGACCCCGACAGCTTGCGCATGCTGCTCACGCAGATGAAC	probe GALA3
RSp0028A	TTTCAGTGAATCGGCACGGCCCGGTCGTGCCCCAGGATGTCGGCCCACGGTCGGCTTCAGCGGACGTG	probe GALA3
RSp0028B	GGAAGTCGGCATCCGGCAATTGATCGTCAAGACCCCGGAGGGTCTCGCGGCGGTCAAGCGCCCAGGCAGC	probe GALA3
RSp0028C	TTTCGGTGAATCGGGCCAACCCGGTCATGGCCCAGGATGTCAGCCCACCGCCGGTTCCGGCGGACGCGC	probe GALA3
RSp0028D	TCGACGCCTGAGGGCCTTGCGGCGGTCAAGCGCGCAGGCAACTATCCGGTCCTGAAAGCGCTGACGCTCG	probe GALA3
RSc1800	CAGGCCCTGGCTGCCAACGATACGCTCGTCACCCTGGATGCCAGCGCCAACTGGATCGGAGACCCGGGCG	probe GALA4
RSc1800A	CGGAGATCTGGACGGAAATCGCGCGCGGCACCAAGCCCGCGGACATGCAAAGCCTGCGCGCCGTCAGCCG	probe GALA4
RSc1800B	GCTCGGGCACTGGCCGCCAGCCAGACGCTCACCCGACTGGACCTGCGCTACAACGAGATCGGGGTCG	probe GALA4

Table S2 (continued): Oligonucleotides used in this study

CGH microarray probes (continued)		
RSc1800C	GGAGATGTGGATGGAAGTCGCGCTCAAGTCCGAGCCTGCGGCCGTTCAACAGATGCGCGCGGTCAGCAAG	probe GALA4
RSc1800D	CCGCATTCGCGCGCAACAAGAAGCTGACCACCCTCAACGTCAGCAGCAACGGGATCGGGCCGGTGGGGGT	probe GALA4
RSc1801	GGTGTCGCTCGACCTGCGCAACAACAGGATGGAAGAATCTGGTACGCGAGCGCTGCTGGCCAACCGCACG	probe GALA5
RSc1801A	TTTTGCGCGCGGGCAACTATCCGGCCCTGGAGAAGCTGACGCTTGCCGGCACGTTCACGGACGACGATCT	probe GALA5
RSc1801B	AACACGAAGCTGACCACGCTCGATCTCGGCTACAACGACATCGGCGATGCCGGCGTGCGGGCGTTGAGCG	probe GALA5
RSc1801C	GTCAAGCGCGCCGGCAACTATCCGGCCCTGGAGAAACTCACGCTCGCCGGCGCTTTCACGGACGCTGACT	probe GALA5
RSc1801D	GCACTGGCCGCCAATACGAGGCTGGCATCGCTCGACCTGCGCAACAACCGGATGCTGGAAGCGGGTGTGC	probe GALA5
RSc1356	GCGCGCAATGTCGTGCTCCAATCTCTCAATCTGAGCTACAACCCCATCGGCTTCTGGGGCGTCAACGCTC	probe GALA6
RSc1356A	GTACCCGGCGCTCAAGAGCGTGCGCTTCAAGGGGGAGCTCACGCTCGAGGCGCTGAAAGCCTTGCCGCCA	probe GALA6
RSc1356B	TCTCGCTCAATCTGCACAACAACGAGATCGGCAACGAGGGCGCGCTGGTGCTGGCTACAAGCCGGACGCT	probe GALA6
RSc1356C	GCGCCAAGGTGCTGGAGGCCAATACCCGGATCACGGGCACGCCGCAGAACCCGAATTTCCTGGCGGAGGA	probe GALA6
RSc1356D	CGGTGCGCAGCAATTGGCGAAAAGCGCCACGTTGACCGAGCTCGACCTGAGCGAGAACCGCATCGGTCCC	probe GALA6
RSc1357	CGGCGCGACAGCAGGAGCAAGACGAGTTTGACACGACGGCCAACGAAATCACAGAGAACGGGACGCGGGC	probe GALA7
RSc1357A	GGTCAGCACCACCATGAATGATGCCGCCCGATCGAGCCAGACGCACATGCAGGCCTGGAACAAGGCGATG	probe GALA7
RSc1357B	CAAGGCGATGCTCGGCCAACTGCATCGCTATCCCAACCTGCAAAGCCTGCGTCTGCGGGGCGACATCACG	probe GALA7
RSc1357C	ATCTGGCGACCAGCGAGTCGATCCAGACGCTTCGCCTGTGCTGCTGCGGTGTCACCGATCCCGGCATCCA	probe GALA7

Table S3: GALA accession numbers and position of *hrp* $_{II}$ motif in *GALA* promoters

GALA names	accession	hrpII box position from atg
**IP01609_GALA2**	CAQ59390	91
IP01609_GALA3	CAQ58784	99
IP01609_GALA4	JF801741	341
IP01609_GALA5	CAQ61423	NA
IP01609_GALA6	CAQ61617	93
IP01609_GALA7	CAQ61616	93
CFBP2957_GALA2	CBJ53541	91
CFBP2957_GALA3	CBJ52829	99
CFBP2957_GALA4	CBJ43095	88
CFBP2957_GALA5	CBJ43096	NA
CFBP2957_GALA6	CBJ43268	412
CFBP2957_GALA7	CBJ43267	96
CMR15_GALA1	CBJ39793	152
**CMR15_GALA2**	CBJ40290	91
CMR15_GALA3	CBJ39687	101
CMR15_GALA4	CBJ38006	372
CMR15_GALA5	CBJ38007	NA
CMR15_GALA6	CBJ38610	97
CMR15_GALA7	CBJ38611	96
CMR15_GALA8	CBJ36437	ND
GMI1000_GALA1	CAD18065	NA
GMI1000_GALA2	CAD17823	91; 228
GMI1000_GALA3	CAD17179	101
GMI1000_GALA4	CAD15502	93
GMI1000_GALA5	CAD15503	NA
GMI1000_GALA6	CAD15058	97
GMI1000_GALA7	CAD15059	93
Molk2_GALA2	CAQ36581	91
Molk2_GALA3	CAQ18416	99
Molk2_GALA4	CAQ35674	88
Molk2_GALA5	CAQ35673	NA
Molk2_GALA6	CAQ18765	72
Molk2_GALA7	CAQ35472	93
PSI07_GALA1-1	CBJ35381	135
PSI07_GALA1-2	CBJ35382	NA
PSI07_GALA1-3	RPSI07_mp0894	NA
PSI07_GALA2	CBJ35242	ND
PSI07_GALA3	CBJ34399	393
PSI07_GALA4	RPSI07_1840	138
PSI07_GALA5	CBJ51205	NA
PSI07_GALA6	CBJ51374	93
PSI07_GALA7	CBJ51373	97
RS1000_GALA1	BAD42410	ND
RS1000_GALA2	BAD42399	ND
RS1000_GALA3	BAH47287	ND
RS1000_GALA4	BAD42394	ND
RS1000_GALA5	BAD42395	NA
RS1000_GALA6	BAD42392	ND
RS1000_GALA7	BAD42393	93

NA: not applicable

ND: not detected

Table S4: Comparative Genomic Hybridization analysis of *GALA* distribution[a] in 60 *R. solanacearum* strains

Phylotype	Strain	GALA1	GALA2	GALA3	GALA4	GALA5	GALA6	GALA7
**IV**	ACH732	0	0	0	1	1	1	0
	Psi07[b]	1	1	1	1	1	1	1
	MAFF301558	0	0	1	1	1	0	1
I	R288	0	1	1	1	1	1	0
	PSS219	1	1	1	1	1	1	1
	PSS81	1	1	1	1	1	1	1
	JT519	0	1	1	1	1	1	0
	JT519	1	1	1	1	1	1	1
	GMI1000[b]	1	1	1	1	1	1	1
	CMR134	1	1	1	1	1	1	0
	PSS358	1	1	1	1	1	1	1
	PSS190	1	1	1	1	1	1	1
III	CFBP3059	0	1	1	1	1	1	0
	CMR15[b]	0	1	1	1	1	1	0
	CMR32	0	1	1	1	1	1	0
	NCPPB1029	0	1	1	1	1	1	1
	CFBP734	0	1	1	1	1	1	1
	J25	0	1	1	1	1	0	1
	NCPPB342	0	1	1	1	1	0	1
	NCPPB332	0	1	1	1	1	1	1
	JT528	0	1	1	1	1	0	1
	JT525	0	1	1	1	1	0	1
	CMR66	0	1	1	1	1	1	1
	CIP358	0	1	1	1	1	1	1
	CMR43	0	1	1	1	1	1	1
II_A	ICMP7963	0	0	1	1	1	0	1
	ISBSF1900	0	1	1	1	1	1	0
	CMR39	0	1	1	1	1	1	1
	UW21	0	1	1	1	1	1	0
	CFBP2957[b]	0	1	1	1	1	1	1
	CIP301	0	1	1	1	1	1	0
	CIP239	0	1	1	1	1	1	0
	B34	0	1	1	1	1	1	1
	A3909	0	1	1	1	0	1	1
II_B	CFBP7014	0	1	1	1	1	1	1
	ISBSF1503	0	1	1	1	1	1	1
	NCPPB3987	0	1	1	1	1	1	1
	ISBSF1712	0	1	1	1	1	1	1
	CIP10	0	1	1	1	1	1	1
	Molk2[b]	0	1	1	1	1	1	1
	CIP418	0	1	1	1	1	1	1
	UW163	0	1	1	1	1	1	1
	Ant307	0	1	1	1	1	1	1
	Ant80	0	1	1	1	1	1	1
	Ant75	0	1	1	1	1	1	1
	JY201	0	1	1	1	1	1	1
	JY200	0	1	1	1	1	1	1
	Ant1121	0	1	1	1	1	1	1
	IPO1609[b]	0	1	1	1	1	1	1
	JT516	0	1	1	1	1	1	1
	CMR34	0	1	1	1	1	1	1
	RE	0	1	1	1	1	1	1
	AP42H	0	1	1	1	1	1	1
	TB1H	0	1	1	1	1	1	1
	TB2H	0	1	1	1	1	1	1
	TC1H	0	1	1	1	1	1	1
	AP31H	0	1	1	1	1	1	1
	TB10	0	1	1	1	1	1	1
	ETAC	0	1	1	1	1	1	1
	RM	0	1	1	1	1	1	1

[a] Presence/absence analysis: 1 stands for presence whereas 0 stands for absence

[b] In red: strains for which whole genome sequence is available
